# Comprehensive analysis of chemokine gene polymorphisms in Korean children with autoimmune thyroid disease

**DOI:** 10.1038/s41598-023-42021-4

**Published:** 2023-09-20

**Authors:** Chungwoo Shin, In-Cheol Baek, Won Kyoung Cho, Tai-Gyu Kim, Byung-Kyu Suh

**Affiliations:** 1 Department of Pediatrics, College of Medicine, St. Vincent’s Hospital, The Catholic University of Korea, Seoul, 065941 Korea; 2 Catholic Hematopoietic Stem Cell Bank, College of Medicine, The Catholic University of Korea, Seoul, 065941 Korea; 3 Department of Microbiology, College of Medicine, The Catholic University of Korea, Seoul, 065941 Korea

**Keywords:** Genetics, Immunology, Endocrinology

## Abstract

Chemokines are chemotactic cytokines that can cause directed migration of leukocytes. The aim of this study was to examine differences in single nucleotide polymorphisms (SNP) of chemokine in AITD patients compared to normal controls. A total of 86 Korean pediatric patients were included in the patient group and 183 adults were included in the normal control group. To compare influences of several chemokine gene polymorphisms, 25 SNPs in 16 chemokine genes were analyzed. Genotype frequencies of CCL11(rs3744508)AA(OR = 6.9) and CCR2(rs1799864)AA(OR = 3.8) were higher in the AITD patients than in the controls, whereas CCL17(rs223828)CC was lower in the AITD patients than in the controls(OR = 0.4). In comparison between Graves' disease (GD) patients and controls, genotype frequency of CCL17(rs223828)CC(OR = 0.4) was lower in the GD group, whereas those of CCR2(rs1799864)AA(OR = 4.8) were higher in the GD group. The genotype frequency of CCL11(rs3744508)AA(OR = 11.3) was higher in Hashimoto's thyroiditis (HT) patients, whereas that of CXCL8(rs2227306)CC(OR = 0.4) was lower in HT patients. Polymorphisms of CCL11(rs3744508), CCL17(rs223828), and CCR2(rs1799864) might be associated with AITD, with CCL17(rs223828), CCR2(rs1799864) and CXCR2(rs2230054, rs1126579) affecting GD and CCL11(rs3744508) and CXCL8(rs2227306) affecting HT in Korean children.

## Introduction

Chemokines are small, chemotactic cytokines that play a crucial role in regulating cell trafficking of various types of leukocytes in development, homeostasis, and inflammation by binding to specific receptors^1,2^. In addition to guiding immune cells to infected or inflamed sites, chemokines coordinate interactions between immune cells, making them a crucial part of various diseases such as infections, cancer, healing recovery, angiogenesis, and autoimmunity^2,3^. Chemokines are divided into four subfamilies (CC, CXC, CX3C, and XC) based on their cysteine configurations, while chemokine receptors are transmembrane receptors that can induce cell migration, adhesion, and other biological responses^1,3,4^. There are also chemokine antagonists being used or studied for treatment^5^.

Autoimmune thyroid disease (AITD) encompasses a range of conditions where the immune system targets the thyroid gland. Classic AITD includes Graves’ disease (GD) and Hashimoto's thyroiditis (HT), both characterized by clinical manifestations of hyperthyroidism in GD and hypothyroidism in HT, along with thyroid infiltration, and the production of thyroid autoantibodies by T and B cells that react with thyroid antigens^6^. AITD may develop when genetically susceptible individuals are exposed to environmental triggers such as infection, iodine, and stress^7,8^. Thyroid associated ophthalmopathy (TAO) is an autoimmune disease that affects ocular and peripheral tissues due to shared autoantigen^9^. Although the recruitment and maintenance of lymphocytes in the thyroid gland is not yet fully understood, considerable clinical and experimental evidence supports the role of lymphocytes in triggering and maintaining AITD^10^. Early onset AITD is likely more strongly influenced by genetic susceptibility than late onset^11^.

AITD stands out as the most prevalent among autoimmune disorders, impacting 15 out of every 1000 individuals in the United States^12^. In a meta-analysis of thyroid dysfunction incidence and prevalence in Europe, it was revealed that the yearly incidence rate for hyperthyroidism stood at 0.51 cases per 1000 individuals within the population^13^. Based on a nationwide population-based cohort study in Korea that focused on the annual incidence and prevalence of thyroid disease, it was identified that the prevalence of individuals undergoing treatment for hyperthyroidism was 2.76 per 1000 people and the annual incidence of new diagnoses for hyperthyroidism, with corresponding ongoing treatment, was found to be 0.55 cases per 1000 individuals in the year 2015^14^.

A number of studies have investigated the relationship between chemokines and AITD, given the important role of chemokines in autoimmune processes^15–17^. Our study aims to determine the relationship between chemokine and AITD by analyzing 25 Single nucleotide polymorphisms (SNPs) of 16 chemokine genes in Korean pediatric AITD patients, and comprehensively examining the associations of these SNPs with different groups of AITD.

## Materials and methods

### Subjects

The present study was conducted on 86 patients diagnosed with AITD [36 with HT and 50 with GD (intractable GD, n = 30) (TAO, n = 24; non-TAO, n = 26)] who were treated at the Pediatric Endocrine Clinics of Seoul St. Mary’s Hospital between March 2009 and August 2021. The age of patients in the study was 13.2 ± 3.3 years at enrollment, and 11.3 ± 3.2 years at diagnosis of AITD (Table 1).

**Table 1:** Characteristics of 86 autoimmune thyroid disease (AITD) patients and controls.

	AITD (n = 86)		Controls (n = 183)
	GD (n = 50)	HT (n = 36)	
Female, n (%)	38 (76.0%)	31 (86.1%)	96 (52.5%)
Age at enrollment (years)	13.2 ± 3.3	13.1 ± 3.9	29.9 ± 3.7
Age at diagnosis (years)	11.3 ± 3.2	10.5 ± 2.7	
Goiter, n (%)	35 (70.0%)	20 (55.6%)	
T3 at diagnosis, 0.78–1.82 ng/mL	3.32 ± 2.00	1.47 ± 0.46	
Free T4 at diagnosis, 0.85–1.86 ng/dL	2.94 ± 1.74	1.30 ± 0.6	
TSH at diagnosis, 0.17–4.05 mIU/L	0.57 ± 2.37	25.93 ± 66.9	
TSHR Ab positive at diagnosis	49 (98.0%)	13 (36.1%)	
Tg Ab positive at diagnosis	28 (56.0%)	28 (77.8%)	
TPO Ab positive at diagnosis	37 (74.0%)	19 (52.8%)	
Clinically evident TAO (NOSPECS class II or higher), n (%)	24 (48.0%)		
Intractable, n (%)	30 (60.0%)		

Data are presented as Mean ± SD or n (%).

AITD, autoimmune thyroid diseases; GD, Graves’ disease; HT, Hashimoto’s thyroiditis; TSH, thyroid Stimulating Hormone; TSHR Ab, TSH receptor antibody; TPO, Thyroid Peroxidase; TAO, thyroid associated ophthalmopathy.

For the control group, a total of 183 healthy Korean adults (52.5% females and 47.5% males; average age: 29.9 ± 3.7 years) without a history of AITD comprising staff and students from the College of Medicine at the Catholic University of Korea were included. All participants provided an informed consent for a genetic study. This study was approved by the Institutional Review Board (IRB) of the Catholic University of Korea (IRB Number: KC09FISI0042, MC13SISI0126), Seoul, Korea, and the study was conducted in accordance with the Declaration of Helsinki.

GD was diagnosed based on clinical symptoms and biochemical confirmation of hyperthyroidism, including diagnosis of goiter, elevated radioactive iodine uptake, antibodies against the thyroid-stimulating hormone (TSH) receptor, and elevated thyroid hormone levels. Patients with other forms of autoimmune diseases, hematologic diseases, or endocrine diseases were excluded. TAO was diagnosed based on the presence of typical clinical features. It was classified according to the system recommended by the American Thyroid Association (ATA) Committee^18^. Patients with no symptoms or the lid lag sign only were included in the non-TAO group, whereas those with soft tissue changes, proptosis, extraocular muscle dysfunction, or the latter two were considered to have an eye disease^19^. In GD, a remission was defined as consistent with improvement of clinical features and restoration of euthyroidism or induction of hypothyroidism after antithyroid drug (ATD) therapy. We defined intractable GD as hyperthyroidism persistent over two years of ATD therapy, relapse after ATD withdrawal, or treatment with ATD for at least 5 years^20–22^. HT was diagnosed when at least three of the following criteria established by Fisher et al.^23^ were met: (1) goiter, diffuse goiter, and decreased radionuclide uptake during thyroid scan, (2) circulating thyroglobulin or microsomal autoantibodies, and (3) hormonal evidence of hypothyroidism.

### DNA extraction

Genomic DNA was extracted from 4 ml of peripheral blood mixed with ethylenediaminetetra acetic acid (EDTA) using an AccuPrep DNA extraction Kit (Bioneer, Daejeon, Republic of Korea): After 20 μl of proteinase K and 200 μl of lysis buffer (200 mM Tris–HCl, 25 mM EDTA, 300 mM NaCl, 1.2% sodium dodecyl sulfate) were added to peripheral blood leukocytes, the mixture was incubated at 60 °C for 10 min. The lysate was extracted with an equal volume of isopropanol. After washing with 500 μl of washing buffer and ethanol, the pellet was dried and suspended in 200 μl of sterile distilled water. DNA extracts were stored at − 20 °C.

### Target gene primer design and polymerase chain reaction (PCR)

Genomic DNA was obtained from multiple samples and the pediatric patient and control groups with AITD were suitable for analysis using a PCR template of 50 ng or lower. The primers used in this study are presented in Table 2. PCR amplifications were performed in 50 µl of reaction mixtures in 96-well thin walled trays (Nippon Genetics, Tokyo, Japan). The reaction mixtures consisted of 10 μmol/l target-specific primers, 150–300 ng genomic DNA, 1 × buffer (60 mmol/l Tris–Cl, 15 mmol/l ammonium sulfate, and 100 mmol/l MgCl2), 250 μmol/l dNTPs (dATP/dGTP/dCTP/dTTP, 250 μmol/l), and 1 U Taq DNA polymerase (Bioprince, Enzynomics, Daejeon, Korea)^24^. In PCR, the extracted genomic DNA was amplified in a ProFlex 96-Well PCR System (Thermo Fisher Scientific, Waltham, MA, USA) using the following PCR conditions: a preliminary step of 1 cycle at 95 °C for 15 min followed by 40 cycles of denaturation at 94 °C for 30 s, annealing at 63 °C for 90 s, and extension at 72 °C for 30 s. The final extension was performed at 60 °C for 30 min^25^.

**Table 2:** Gene information.

Gene(position	rs number	SNP (Wild/Variant allele)^§^	Chromosome number	SNP position	Direction	Sequence (5′–3′)	Specific sequence			
							Span (bp)*	Tm	Length	Amplicon size (bp)
CCL2(−2518)	rs1024611	A/G	chr17	32579788	Forward	ACAGGGAAGGTGAAGGGTATGAATCA	50	76	26	110
		(A>G)			Reverse	GGGGAGGGCATCTTTTCTTGACA		70	23	
CCL2(−2076)	rs1024610	T/A	chr17	32580231	Forward	TCACCTCATCTTCCAAGAAGTTTTCCTC	51	80	28	110
		(T>A,G)			Reverse	GGAAATTGAATGCGGTCCACCA		66	22	
CCL5(−403)	rs2107538	G/A	chr17	34207780	Forward	CTCACTGAGTCACTGAGTCTTCAAAGTTCC	59	88	30	110
		(C> T)			Reverse	CCAGAGGACCCTCCTCAATAAAACACT		80	27	
CCL5(+375)	rs2280789	T/C	chr17	34207003	Forward	CTCTATGCTGCTTCATGGCAGGG	50	72	23	110
		(A>C,G,T)			Reverse	AACATGAGTCCACACTCAGTGAACACC		80	27	
CCL5(−28)	rs2280788	G/C	chr17	34207405	Forward	GGCCAATGCTTGGTTGCTATTTTG	50	70	24	110
		(G > C)			Reverse	CTCTGCAGGAATCCTCTGCAGCTC		76	24	
CCL11(+67)	rs3744508	G/A	chr17	32612894	Forward	ACTTCTGTGGCTGCTGCTCATAGC	50	74	24	110
		(G>A,C,T)			Reverse	GGAGGTGGTTACCTTACCTTTCCTGTAAG		86	29	
CCL17(−431)	rs223828	T/C	chr16	57447414	Forward	GAAGCTCACTCATGGTCCAGGAGAA	50	76	25	110
		(T>A,C)			Reverse	ATGCATGGTGGCTTGAGCAGG		66	21	
CCL24(+179)	rs2302004	T/C	chr7	75442855	Forward	TGCAGGACTGACCAGCCTGG	51	66	20	110
		(A>G,T)			Reverse	GGAGCTTGAACCCTGCACCAAA		68	22	
CCL24(+275)	rs2302005	C/T	chr7	75442759	Forward	AGCTCTGAGGCCCAGAGCCAA	51	68	21	110
		(G>A,C)			Reverse	AGCAGGGAGAGGGGATGACCA		68	21	
CCL26(+2497)	rs2302009	T/G	chr7	75769680	Forward	TGGGTGCAAAAATACATTTCTTTACTGAAA	55	78	30	110
		(A>C,G,T)			Reverse	CAGAGGGCTGCAGAGCCAAGA		68	21	
CXCL5(−156)	rs352046	G/C	chr4	74864550	Forward	CGTCCCTCTCGGTAGAGGTGCA	50	72	22	110
		(G> A,C,T)			Reverse	TGTGGAAAGAAGAGGTTGGGGGA		70	23	
CXCL8(−251)	rs4073	A/T	chr4	74606024	Forward	CTGCCACTCTAGTACTATATCTGTCACATGG	49	90	31	110
		(A>C,G,T)			Reverse	CATTTAAAATACTGAAGCTCCACAATTTGG		80	30	
CXCL8(+781)	rs2227306	C/T	chr4	74607055	Forward	CCATGAAGATGTTGATATTGTACAAAAAGAAC	52	84	32	110
		(C>T)			Reverse	GCCCTTGACCTCAGTTAGTTCTTTGTTTT		82	29	
CXCL9(intron1)	rs2276886	C/T	chr4	76928428	Forward	TAGCCAGATAAAGGTGAATGTTCTGCC	53	78	27	110
		(C>A,T)			Reverse	GGCAGATCGAAGTTATGAACTTTGGATAAC		84	30	
CXCL10(+1000)	rs8878	A/G	chr4	76942300	Forward	CAAAATAAAAATGAGGTACTCTCCTGGAAA	56	80	30	110
		(A>C,G,T)			Reverse	CAAGTGACACACAAGGCACTTCATCTTAG		84	29	
CXCL10(+1642)	rs3921	C/G	chr4	76942943	Forward	GGATGATGAACATTAACCTTCCTACAGGAG	60	86	30	110
		(C>A,G,T)			Reverse	CCTACATGGAGTATATGTCAAGCCATAATTG		86	31	
CXCL12(+801)	rs1801157	G/A	chr10	44868257	Forward	GAGGGCCACATGGGAGGCTC	49	68	20	110
		(C>T)			Reverse	CTCAGCTCAGGGTAGCCCTGCT		72	22	
CCR2(+180)	rs1799864	G/A	chr3	46399208	Forward	CGCTCTACTCGCTGGTNTTCATCTTT	49	74	26	110
		(G>A)			Reverse	CAGGTAAATGTCAGTCAAGCACTTCAGC		82	28	
CCR5(−59,029)	rs1799987	A/G	chr3	46411935	Forward	GGTTGGGGTGGGATAGGGGATA	49	70	22	110
		(A>G)			Reverse	AACTTAAACCAACTTTAAATGTAGAGGGGG		82	30	
CCR5(+59,353)	rs1799988	C/T	chr3	46412259	Forward	CAAAATAATCCAGTGAGAAAAGCCCG	57	74	26	110
		(C>T)			Reverse	GGCGAAAAGAATCAGAGAACAGTTCTTCT		82	29	
CXCR2(+785)	rs2230054	C/T	chr2	219000310	Forward	GGGCCATGCGGGTCATCTTT	50	64	20	110
		(C>T)			Reverse	GTCCTCATGAGGGTGTCTGCCAG		74	23	
CXCR2(+1208)	rs1126579	T/C	chr2	219000734	Forward	GGTTCTTCTTGGTCTCAGTGTCAATGC	49	80	27	110
		(T>C)			Reverse	CAAGCTTTCTAAACCATGCAAGGGA		72	25	
CX3CR1(+652)	rs3732378	G/A	chr3	39307162	Forward	CCCAGCAAATGCATAGATGAGAGGAT	51	76	26	110
		(G>A)			Reverse	CCAGTTGTGACATGAGGAAGGATCTG		78	26	
CX3CR1(+745)	rs3732379	C/T	chr3	39307256	Forward	GTCACAACTGGGAAAGAAGTCATAGAGCT	51	84	29	110
		(C>T)			Reverse	ACTGATCCTTCTGGTGGTCATCGTG		76	25	
CXCR1(−2668)	rs2671222	A/G	chr2	219032602	Forward	ACAGCCATTAGCTGCTGGAGGC	51	70	22	110
		(T>C)			Reverse	GGAAGAGCTGTGGTCAAGGTCACTG		78	25	
Universal Primer						GATCAGGCGTCTGTCGTGCTC		58	21	

^§^SNP information referred from dbSNP (https://www.ncbi.nlm.nih.gov/snp).

*Span: The distance between forward and reverse primers on target sequence.

Universal primer: The universal sequences were not matched with any genomic DNA sequences. All the primers include a universal sequence at individual 5′-end.

### Sequencing

Sanger sequencing was performed using a Big Dye Terminator v3.1 (Amersham Pharmacia) and reactions were analyzed with ABI PRISM 3730XL analyzer (PE Applied Biosystems, Foster City, CA, USA). Sequencing data was analyzed using the FinchTV 1.4 software (Geospiza, Inc., Seattle, Washington, USA).

### Statistical analysis

The allele frequencies were calculated using Microsoft Office Excel. The statistical significance was assessed using the χ^2^ test and Fisher’s exact test. The p values were adjusted by the Bonferroni method and OR was calculated using Haldane’s modification of Woolf’s method^26,27^. A two-tailed p value < 0.05 was considered statistically significant. The Hardy–Weinberg equilibrium (HWE) of each SNPs were evaluated using the Haploview software version 4.2^28^ and all SNPs fit HWE. The power of our study was calculated based on an available sample size of 50 GD cases and 186 controls. The power for rs223828 minor allele frequency (MAF) (MAF: 25.6%) was 0.75 when OR was 0.4. Sample size and power were calculated using Quanto software version 1.2.4 (University of Southern California; Los Angeles, CA, USA).

## Results

### Comparison of genotype and allele frequencies of 25 SNPs of 16 chemokine genes in AITD patients and controls

An analysis of the genotype of the SNP in the entire patient group, including those with GD and HT is shown in Table 3. Genotype frequencies of CCL11(rs3744508) AA (7.1% vs. 1.1%, OR = 6.9, Pc = 0.023) and CCR2(rs1799864) AA (16.3% vs. 4.9%, OR = 3.8, Pc = 0.006) were higher in the total AITD patent group than in the control group, whereas that of CCL17(rs223828) CC (21.4% vs. 39.3%, OR = 0.4, Pc = 0.012) was lower in the total AITD patient group than in the control group. Allele frequency of CCL17(rs223828) T (51.6% vs. 42.2%, OR = 1.5, Pc = 0.041) was higher in the total AITD patient group than in the control group. Other SNPs were not statistically significant between AITD patients and controls (Table 4).

**Table 3:** Genotype frequency.

Gene	rs number	SNP	Genotype	Control	n = 183	AITD (GD + HT)	n = 86
		(Wild/Variant allele)^§^		n	%	n	%
CCL2	rs1024611	−2518 A/G	GG	69	37.7	40	46.5
		(A > G)	GA	89	48.6	36	41.9
			AA	25	13.7	10	11.6
	rs1024610	−2076 T>A	AA	158	86.3	74	92.5
		(T>A,G)	AT	25	13.7	5	6.3
			TT	0	0	1	1.3
CCL5	rs2107538	−403 G>A	GG	73	39.9	34	40
		(C>T)	GA	76	41.5	39	45.9
			AA	34	18.6	12	14.1
	rs2280789	+375 T/C	CC	31	16.9	11	12.8
		(A>C,G,T)	CT	77	42.1	40	46.5
			TT	75	41.0	35	40.7
	rs2280788	−28 G/C	GG	125	68.3	61	71.8
		(G>C)	CG	50	27.3	22	25.9
			CC	8	4.4	2	2.4
CCL11	rs3744508	+67 G/A	GG	125	68.3	58	68.2
	(= rs1129844)	(G>A,G,T)	GA	56	30.6	21	24.7
			AA	2	1.1	6	7.1 a
CCL17	rs223828	−431 T/C	CC	72	39.3	18	21.4 b
		(T>A,C)	CT	80	43.7	44	52.4
			TT	31	16.9	22	26.2
CCL24	rs2302004	+179 T/C	CC	82	44.8	41	47.7
		(A>G,T)	CT	81	44.3	36	41.9
			TT	20	10.9	9	10.5
	rs2302005	+275 C/T	CC	25	13.7	10	11.9
		(G>A,C)	CT	79	43.2	38	45.2
			TT	79	43.2	36	42.9
CCL26	rs2302009	+2497 T/G	GG	3	1.6	2	2.3
		(A>C,G,T)	GT	26	14.2	18	20.9
			TT	154	84.2	66	76.7
CXCL5	rs352046	−156 G/C	GG	0	0.0	0	0.0
		(G>A,C,T)	GC	7	3.8	2	2.4
			CC	176	96.2	83	97.6
CXCL8	rs4073	−251 A/T	AA	24	13.1	14	16.5
		(A>C,G,T)	AT	83	45.4	40	47.1
			TT	76	41.5	31	36.5
	rs2227306	+781 C/T	CC	90	49.2	36	42.4
		(C>T)	CT	74	40.4	39	45.9
			TT	19	10.4	10	11.8
CXCL9	rs2276886	intron1 C/T	CC	63	34.4	33	38.8
		(C>A,T)	CT	90	49.2	45	52.9
			TT	30	16.4	9	8.2
CXCL10	rs8878	+1000 G/A	GG	167	91.3	82	96.5
		(A>C,G,T)	GA	15	8.2	3	3.5
			AA	1	0.5	0	0
	rs3921	+1642 C/G	GG	167	91.3	82	95.3
		(C>A,G,T)	GC	15	8.2	4	4.7
			CC	1	0.5	0	0
CXCL12	rs1801157	+801 G/A	GG	100	54.6	48	56.5
		(C>T)	GA	72	39.3	30	35.3
			AA	11	6.0	7	8.2
CCR2	rs1799864	+180 G/A	GG	90	49.2	37	43
		(G>A)	GA	84	45.9	35	40.7
			AA	9	4.9	14	16.3 c
CCR5	rs1799987	−59029 A/G	GG	51	27.9	22	25.9
		(A>G)	GA	94	51.4	42	49.4
			AA	38	20.8	21	24.7
	rs1799988	+59353 C/T	CC	38	20.8	21	24.7
		(C>T)	CT	94	51.4	42	49.4
			TT	51	27.9	22	25.9
CXCR2	rs2230054	+785 C/T	CC	78	42.6	45	53.6
		(C>T)	CT	81	44.3	28	33.3
			TT	24	13.1	11	13.1
	rs1126579	+1208 T/C	CC	27	14.8	15	17.4
		(T>C)	CT	82	44.8	28	32.6
			TT	74	40.4	43	50
CX3CR1	rs3732378	+652 G/A	GG	166	90.7	77	90.6
		(G>A)	GA	16	8.7	8	9.4
			AA	1	0.5	0	0
	rs3732379	+745 C/T	CC	166	90.7	78	89.5
		(C>T)	CT	16	8.7	9	9.3
			TT	1	0.5	1	1.2
CXCR1	rs2671222	−2668 G/A	GG	146	79.8	73	85.9
		(T>C)	GA	36	19.7	10	11.8
			AA	1	0.5	2	2.4

^§^SNP information referred from dbSNP (https://www.ncbi.nlm.nih.gov/snp).

a. cP = 0.023, OR = 6.9, CI [1.4–34.8].

b. cP = 0.012, OR = 0.4, CI [0.2–0.8].

c. cP = 0.006, OR = 3.8, CI [1.6–9.1].

**Table 4:** Allele frequency.

Gene	rs number	SNP	Allele	Control	n = 183	AITD (GD + HT)	n = 86
		(Wild/Variant allele)^§^		n	%	n	%
CCL2	rs1024611	−2518 A/G	G	158	58.1	76	62.3
		(A>G)	A	114	41.9	46	37.7
	rs1024610	−2076 T>A	A	183	88	79	92.9
		(T>A,G)	T	25	12	6	7.1
CCL5	rs2107538	−403 G>A	G	149	57.5	73	58.9
		(C>T)	A	110	42.5	51	41.1
	rs2280789	+375 T/C	C	108	41.5	51	40.5
		(A>C,G,T)	T	152	58.5	75	59.5
	rs2280788	−28 G/C	G	175	75.1	83	77.6
		(G>C)	C	58	24.9	24	22.4
CCL11	rs3744508	+67 G/A	G	181	75.7	79	74.5
	(= rs1129844)	(G>A,G,T)	A	58	24.3	27	25.4
CCL17	rs223828	−431 T/C	C	152	57.8	62	48.4
		(T>A,C)	T	111	42.2	66	51.6 a
CCL24	rs2302004	+179 T/C	C	163	61.7	77	63.1
		(A>G,T)	T	101	38.3	45	36.9
	rs2302005	+275 C/T	C	104	39.7	48	39.3
		(G>A,C)	T	158	60.3	74	60.7
CCL26	rs2302009	+ 2497 T/G	G	29	13.9	20	19.2
		(A>C,G,T)	T	180	86.1	84	80.8
CXCL5	rs352046	−156 G/C	G	7	3.7	2	2.3
		(G>A,C,T)	C	183	96.3	85	97.7
CXCL8	rs4073	−251 A/T	A	107	40.2	54	43.2
		(A>C,G,T)	T	159	59.8	71	56.8
	rs2227306	+781 C/T	C	164	63.8	75	60.5
		(C>T)	T	93	36.2	49	39.5
CXCL9	rs2276886	intron1 C/T	C	153	56	78	60
		(C>A,T)	T	120	44	52	40
CXCL10	rs8878	+1000 G/A	G	182	91.9	85	96.6
		(A>C,G,T)	A	16	8.1	3	3.4
	rs3921	+1642 C/G	G	182	91.9	86	95.6
		(C>A,G,T)	C	16	8.1	4	4.4
CXCL12	rs1801157	+801 G/A	G	172	67.5	78	67.8
		(C>T)	A	83	32.5	37	32.2
CCR2	rs1799864	+ 180 G/A	G	174	65.2	72	59.5
		(G>A)	A	93	34.8	49	40.5
CCR5	rs1799987	−59029 A/G	G	145	52.3	64	50.4
		(A>G)	A	132	47.7	63	49.6
	rs1799988	+59353 C/T	C	132	47.7	63	49.6
		(C>T)	T	145	52.3	64	50.4
CXCR2	rs2230054	+785 C/T	C	159	60.2	73	65.2
		(C>T)	T	105	39.8	39	34.8
	rs1126579	+1208 T/C	C	109	41.1	43	37.7
		(T>C)	T	156	58.9	71	62.3
CX3CR1	rs3732378	+652 G/A	G	182	91.5	85	91.4
		(G>A)	A	17	8.5	8	8.6
	rs3732379	+745 C/T	C	182	91.5	85	90.4
		(C>T)	T	17	8.5	9	9.6
CXCR1	rs2671222	−2668 G/A	G	182	83.1	83	87.4
		(T>C)	A	37	16.9	12	12.6

^§^SNP information referred from dbSNP (https://www.ncbi.nlm.nih.gov/snp).

a. *cP* = 0.041, OR = 1.5, CI [1.0–2.2].

### Comparison of genotype and allele frequencies of four chemokine genes in GD patients and controls (Table 5)

**Table 5:** GD related genotype and allele.

Gene	rs number	SNP	Genotype/Allele	Control	n = 183	GD	n = 50	GD intractable	n = 30	GD remission	n = 20	TAO	n = 24	Non-TAO	n = 26
		(Wild/Variant allele)^§^		n	%	n	%	n	%	n	%	n	%	n	%
CCL17	rs223828	−431 T/C	CC	72	39.3	10	20.4 a	3	10.0 b	7	36.8	4	16.7 e	6	24.0
		(T>A,C)	CT	80	43.7	25	51	17	56.7	8	42.1	12	50.0	14	56.0
			TT	31	16.9	14	28.6	10	33.3 c	4	21.1	8	33.3	5	20.0
			C	152	57.8	35	47.3	20	42.6	15	55.6	16	44.4	20	51.3
			T	111	42.2	39	52.7	27	57.4 d	12	44.4	20	55.6	19	48.7
CCR2	rs1799864	+180 G/A	GG	90	49.2	22	44.0	14	46.7	8	40.0	12	50.0	10	38.5
		(G>A)	GA	84	45.9	18	36.0	11	36.7	7	35.0	8	33.3	10	38.5
			AA	9	4.9	10	20.0 f	5	16.7 g	5	25.0 h	4	16.7 i	6	23.1 j
			G	174	65.2	40	58.8	25	61.0	15	55.6	20	62.5	20	55.6
			A	93	34.8	28	41.2	16	39.0	12	44.4	12	37.5	16	44.4
CXCR2	rs2230054	+785 C/T	CC	78	42.6	30	61.2 k	18	60.0	12	63.2	13	54.2	17	68.0 l
		(C>T)	CT	81	44.3	15	30.6	8	26.7	7	36.8	8	33.3	7	28.0
			TT	24	13.1	4	8.2	4	13.3	0	0.0	3	12.5	1	4.0
			C	159	60.2	45	70.3	26	68.4	19	73.1	21	65.6	24	75.0
			T	105	39.8	19	29.7	12	31.6	7	26.9	11	34.4	8	25.0
CXCR2	rs1126579	+1208 T/C	CC	27	14.8	5	10.0	5	16.7	0	0.0	4	16.7	1	3.8
		(T>C)	CT	82	44.8	16	32.0	8	26.7	8	40.0	7	29.2	9	34.6
			TT	74	40.4	29	58.0 m	17	56.7	12	60.0	13	54.2	16	61.5 n
			C	109	41.1	21	31.8	13	34.2	8	28.6	11	35.5	10	28.6
			T	156	58.9	45	68.2	25	65.8	20	71.4	20	64.5	25	71.4

^§^SNP information referred from dbSNP (https://www.ncbi.nlm.nih.gov/snp).

a. cP = 0.041, OR = 0.4, CI [0.2–0.8].

b. cP = 0.005, OR = 0.2, CI [0.1–0.6].

c. cP = 0.035, OR = 2.5, CI [1.0–5.7].

d. cP = 0.049, OR = 1.9, CI [1.1–3.3].

e. cP = 0.030, OR = 0.3, CI [0.1–0.9].

f. cP = 0.002, OR = 4.8, CI [1.8–12.7].

g. cP = 0.048, OR = 3.9, CI [1.2–12.5].

h. cP = 0.002, OR = 6.4, CI [1.9–21.7].

i. cP = 0.026, OR = 3.9, CI [1.1–13.7].

j. cP = 0.002, OR = 5.8, CI [1.9–18.0].

k. cP = 0.02, OR = 2.1, CI [1.1–4.1].

l. cP = 0.017, OR = 2.9, CI [1.2–7.0].

m. cP = 0.027, OR = 2.0, CI [1.1–3.8].

n. cP = 0.042, OR = 2.4, CI [1.0–5.5].

In comparison between GD patients and controls, the genotype frequency of CCL17(rs223828) CC (20.4% vs. 39.3%, OR = 0.4, Pc = 0.041) was lower in GD patients, whereas genotype frequencies of while CCR2(rs1799864) AA (20.0% vs. 4.9%, OR = 4.8, Pc = 0.002), CXCR2(rs2230054) CC (61.2% vs. 42.6%, OR = 2.1, Pc = 0.02), and CXCR2(rs1126579) TT (58.0% vs. 40.4%, OR = 2.0, Pc = 0.027) were higher in GD patients. In the intractable GD group, genotype frequencies of CCL17(rs223828) TT (33.3% vs. 16.9%, OR = 2.5, Pc = 0.035), T (57.4% vs. 44.4%, OR = 1.9, Pc = 0.049) and CCR2(rs1799864) AA (16.7% vs. 4.9%, OR = 3.9, Pc = 0.048) were higher, whereas that of CCL17(rs223828) CC (10% vs. 39.3%, OR = 0.2, Pc = 0.005) was lower than in the control. In the TAO group, CCL17(rs223828) CC (16.7% vs. 39.4%, OR = 0.3, Pc = 0.030) had lower frequency, whereas CCR2(rs1799864) AA (16.7% vs. 4.9%, OR = 3.9, Pc = 0.026) had higher frequency than the control group. In the non-TAO group CCR2(rs1799864) AA (23.1% vs. 4.9%, OR = 5.8, Pc = 0.002), CXCR2(rs2230054) CC (68.0% vs. 42.6%, OR = 3.9. Pc = 0.026) and CXCR2(rs1126579) TT (61.5% vs. 40.4%, OR = 2.4, Pc = 0.042) had higher frequencies than the control group.

### Comparison of genotype and allele frequencies of four chemokine genes in HT patients and controls (Table 6)

**Table 6:** HT related genotype and allele.

Gene	rs number	SNP	Genotype/Allele	Control	n = 183	HT	n = 36
		(Wild/Variant allele)^§^		n	%	n	%
CCL11	rs3744508	+67 G/A	GG	125	68.3	23	63.9
	(= rs1129844)	(G>A,G,T)	GA	56	30.6	9	25.0
			AA	2	1.1	4	11.1 a
			G	181	75.7	32	71.1
			A	58	24.3	13	28.9
CXCL8	rs2227306	+781 C/T	CC	90	49.2	10	27.8 b
		(C>T)	CT	74	40.4	20	55.6
			TT	19	10.4	6	16.7
			C	164	63.8	30	53.6
			T	93	36.2	26	46.4

^§^SNP information referred from dbSNP (https://www.ncbi.nlm.nih.gov/snp).

a. cP = 0.002, OR = 11.3, CI [2.0–64.4].

b. cP = 0.018, OR = 0.4, CI [0.2–0.9].

In comparison between control and HT patients, two SNPs showed statistical differences: CCL11(rs3744508) AA (11.1% vs. 1.1%, OR = 11.3, Pc = 0.002) had a higher frequency in HT patients, whereas CXCL8(rs2227306) CC (27.8% vs. 49.2%, OR = 0.4, Pc = 0.018) had lower frequency in HT patients.

## Discussion

In this study, we observed significant differences in the frequencies of SNPs CCL11(rs3744508), CCL17(rs223828), and CCR2(rs1799864) were observed between the control group and AITD in Korean children. The AITD group had a higher T allele frequency in CCL17(rs223828), higher genotype frequencies in CCL11(rs3744508)AA and CCR2(rs1799864)AA, and a lower frequency in CCL17(rs223828)CC compared to controls. Upon dividing AITD into GD and HT, we found significant differences in CCL17(rs223828), CCR2(rs1799864), and CXCR2(rs2230054, rs1126579) between the control group and the GD group. Among GD patients, frequencies of CCR2(rs1799864)AA, CXCR2(rs2230054)CC, and CXCR2(rs1126579)TT were higher, and CCL17(rs223828)CC was lower compared to controls. In HT patients, CCL11(rs3744508)AA was higher, and CXCL8(rs2227306)CC was lower than controls.

Associations between chemokine SNPs and immune-related diseases, including AITD, have been previously documented. The T allele in CCL17(rs223828) was associated with increased serum CCL17 levels as well as increased coronary artery disease risk in a Chinese Han Population^29^. In relation to CCL17, Aso et al.^30^ confirmed with reports of higher levels of CCL17 and CXCL10 in serum in GD patients accompanied by type 1 diabetes. SNPs such as CCL11(rs1129844) have also been connected with elevated plasma chemokine levels in conditions like Fibromyalgia^31^. In the population of India, a significant association was found between SNPs CCR2 (rs1799864A) and Japanese encephalitis^32^, an observation supported by the overexpression of CCL2 (a ligand that can bind to CCR2) in GD^33^. Even though no direct connections have been reported between cxcr2 polymorphism and AITD, research has uncovered associations with other medical conditions. For instance, in China, the CXCR2 (rs2230054) CC genotype occurred more frequently in patients with thoracic aortic aneurysm^34^. Additionally, individuals carrying the CXCR2 rs1126579 TT genotype showed a significant increase in the likelihood of HCV spontaneous clearance^35^. Early studies focusing on chemokines of HT have revealed the role of CXCL8^36^. Weetman et al.^37^ demonstrated that thyroid cells express CXCL8 upon stimulation with inflammatory cytokines such as IFN-r, TNF-α, and IL-1. Interestingly, the CC genotype of CXCL8(rs2227306) was found to be associated with susceptibility to sepsis in males^38^, indicating a broader impact of these polymorphisms on immune function and disease susceptibility.

In addition to the chemokine SNPs significantly identified in our study, various other chemokine SNPs have been associated with GD or HT, underlining the complexity of the relationship between these genetic variations and AITD. Several chemokines, including CCL3, CCL4, CCL21, CXCL9, CXCL10, and CXCL11, have been reported to link specifically to GD and HT^37,39–45^. In the early stage of GD, relationships are seen between chemokines and immune cells. CXCL10, made by follicular epithelial cells in the thyroid, attracts specific immune cells called CXCR3-expressing Th1 cells, leading to inflammation^41^. Research has shown that people with GD have more CXCL10 in their blood than people of the same age and gender without GD^42^. This same pattern has been found with other similar proteins, CXCL9 and CXCL11, where higher levels were found in the blood of people with GD compared to those without the condition^43^. The functional dynamics of chemokines in AITD have also been explored. Other research has examined the interaction of chemokines and other cytokines in primary thyrocyte cultures from GD patients, discovering that PPAR-α ligands can inhibit the secretion of CXCL9, CXCL10, and CXCL11 in a dose-dependent manner^44,45^. This finding provides potential insights into therapeutic pathways. Furthermore, Garcia-Lopez et al. identified increased expression levels of CXCL9 and CXCL10 in the thyroid of HT patients, thus revealing a specific association of CXCL9 and CXCL10 with HT^46^. These observations collectively illuminate the multifaceted role of chemokines in the pathogenesis of AITD, and underscore the need for continued investigation into these complex interactions.

This study uncovered statistically significant differences in the frequencies of SNPs in CCL17, CCR2, and CXCR2 between the GD group and the control group. Additionally, we identified significant differences in the frequencies of SNPs of CCL11 and CXCL8 between the HT group and the control group. These findings not only confirm the association between the CXCL8 gene polymorphism and HT but also suggest that CCL11 might be a potential contributing factor to HT. In practical terms, these insights could lead to novel therapeutic avenues. Targeting chemokines through antagonists, such as CCR4, CCR5, and CXCR4, might present a new strategy for treating AITD and other related conditions. This emphasizes the importance of understanding these genetic variations, as they could provide key information for the development of future treatments.

This study has some limitations. First, the power of statistics is inevitably low because of small number of patients. Second, we did not investigate how the specific genetic variations, or SNPs, we identified influence the levels of chemokines in the patient’s blood. Understanding this relationship could offer useful insights for predicting the disease. Future research should focus on exploring how these SNPs affect serum levels, as this could provide a more comprehensive understanding of the underlying mechanisms in AITD. In conclusion, this study identified six SNPs in five genes (CCL11, CCL17, CCR2, CXCR2, and CXCR8) that may be linked to an increased risk of AITD, GD, and HT in Korean pediatric patients. These findings point to a potential genetic relationship between these specific SNPs and the development of AITD, offering a new perspective on the underlying mechanisms of the disease. This requires larger sample sizes and possibly the inclusion of samples from diverse regions, to build a more comprehensive and definitive understanding of these genetic influences on AITD.

## Data Availability

The datasets generated and/or analysed during the current study are available in the Harvard dataverse repository, https://doi.org/10.7910/DVN/IAWYPJ.
